# Pharmacological inhibitors of β-cell dysfunction and death as therapeutics for diabetes

**DOI:** 10.3389/fendo.2023.1076343

**Published:** 2023-03-15

**Authors:** Stéphane Dalle, Amar Abderrahmani, Eric Renard

**Affiliations:** ^1^ Institut de Génomique Fonctionnelle, Université de Montpellier, Centre National de la Recherche Scientifique (CNRS), Institut National de la Santé et de la Recherche Médicale (INSERM), Montpellier, France; ^2^ Université Lille, Centre National de la Recherche Scientifique (CNRS), Centrale Lille, Polytechnique Hauts-de-France, UMR 8520, IEMN, Lille, France; ^3^ Laboratoire de Thérapie Cellulaire du Diabète, Centre Hospitalier Universitaire, Montpellier, France; ^4^ Département d’Endocrinologie, Diabètologie, Centre Hospitalier Universitaire, Montpellier, France

**Keywords:** diabetes, pancreatic β-cell, insulin secretion dysfunction, apoptosis, pharmacological inhibitors, therapeutic strategies

## Abstract

More than 500 million adults suffer from diabetes worldwide, and this number is constantly increasing. Diabetes causes 5 million deaths per year and huge healthcare costs per year. β-cell death is the major cause of type 1 diabetes. β-cell secretory dysfunction plays a key role in the development of type 2 diabetes. A loss of β-cell mass due to apoptotic death has also been proposed as critical for the pathogenesis of type 2 diabetes. Death of β-cells is caused by multiple factors including pro-inflammatory cytokines, chronic hyperglycemia (glucotoxicity), certain fatty acids at high concentrations (lipotoxicity), reactive oxygen species, endoplasmic reticulum stress, and islet amyloid deposits. Unfortunately, none of the currently available antidiabetic drugs favor the maintenance of endogenous β-cell functional mass, indicating an unmet medical need. Here, we comprehensively review over the last ten years the investigation and identification of molecules of pharmacological interest for protecting β-cells against dysfunction and apoptotic death which could pave the way for the development of innovative therapies for diabetes.

## 1 Introduction

**Figure 1:**
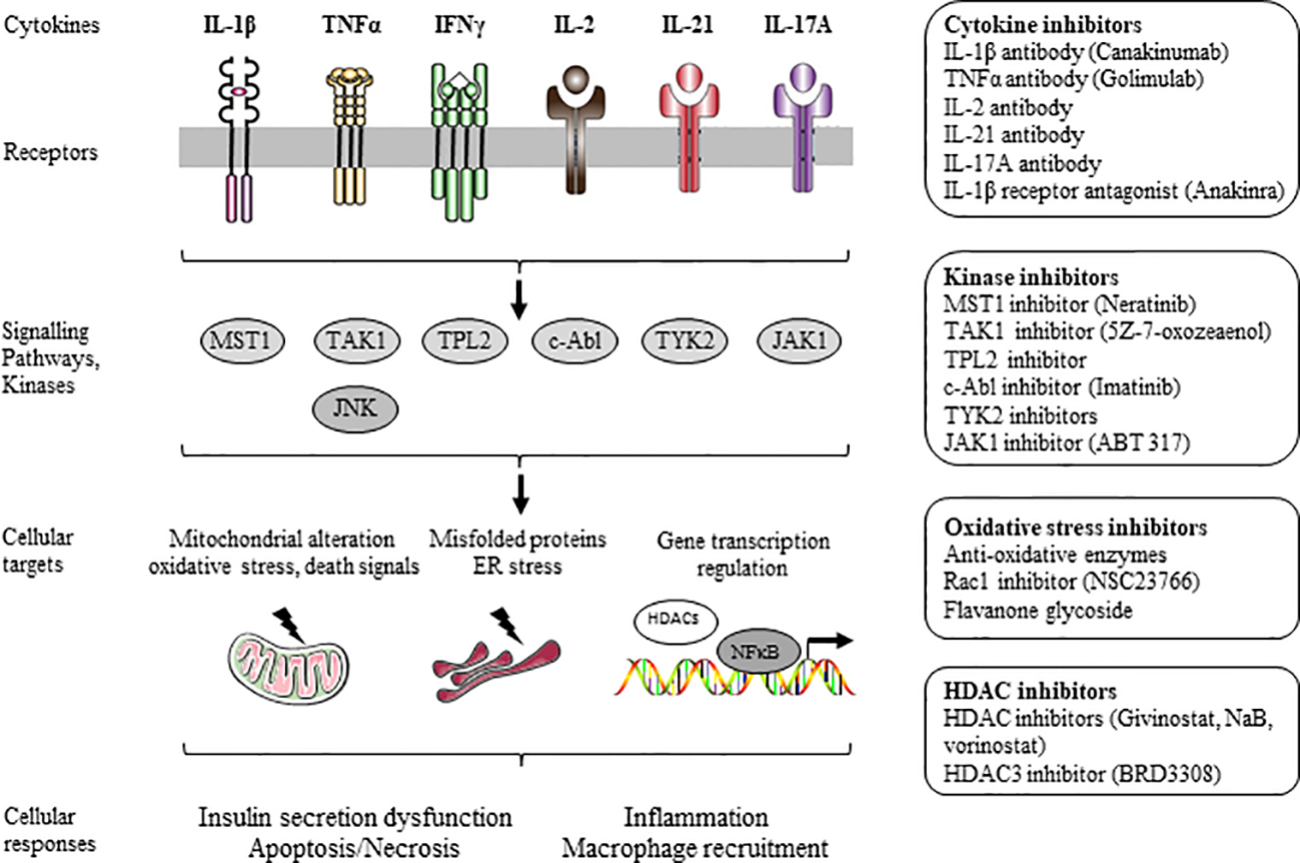
Inhibitors of β-cell dysfunction and death induced by inflammation.

**Figure 2:**
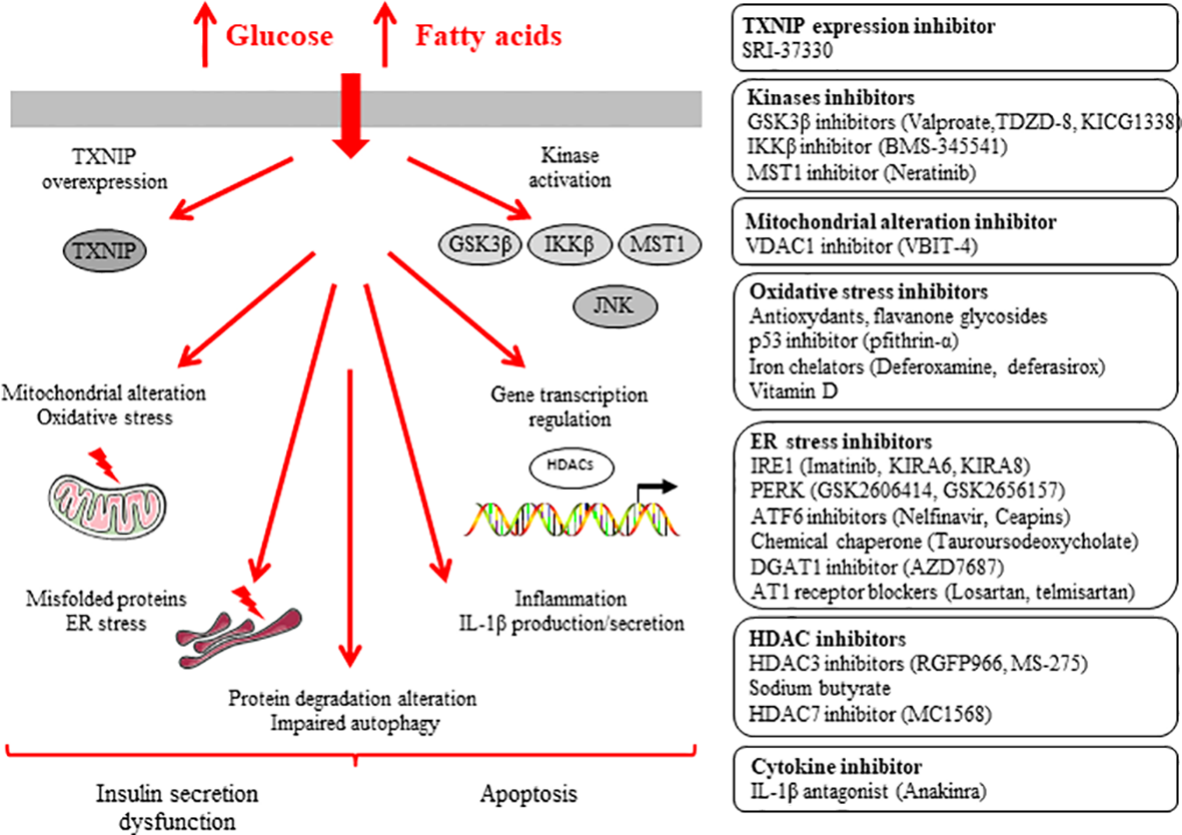
Inhibitors of β-cell dysfunction and death induced by glucotoxicity and glucolipotoxicity.

In type 1 diabetes (T1D), autoimmune destruction of pancreatic β-cells and reduction in β-cell mass are key characteristics of the disease (1–4). The destruction of β-cells is due to immune-mediated processes such as mononuclear infiltration into pancreatic islets leading to elevated intraislet concentrations of pro-inflammatory cytokines and chemokines (1–3). In type 2 diabetes (T2D), β-cell secretory dysfunction is a key event in the development of a clinically evident disease (5–8). A loss of β-cell mass by apoptotic death has also been proposed as critical for the pathogenesis of T2D (9, 10). Dysfunction and death by apoptosis of β-cells in T2D are caused by multiple factors, including chronic hyperglycemia (glucotoxicity), certain high-concentration fatty acids (lipotoxicity), reactive oxygen species (ROS), endoplasmic reticulum (ER) stress, and islet amyloid polypeptide deposits (11–15). Chronic, systemic and low grade inflammation was also proposed to lead to β-cell dysfunction and death, and ultimately to T2D (16, 17). Thus, in both T1D and T2D, a loss of β-cell survival and function contributes to the development of absolute or relative insulin insufficiency. Over the last years, several molecules and drugs have been developed and shown to inhibit β-cell apoptosis and dysfunction through different mechanisms of action (**Figures 1**, **2**). In this review, we comprehensively highlight the development of these molecules and drugs over the last ten years that protect β-cells against apoptotic death and dysfunction which present key features for diabetes therapy (**Table 1**).

**Table 1:** Experimental models used to test and develop pharmacological inhibitors of β-cell dysfunction and death.

Inhibitors of β-cell dysfunction and death	Experimental models and development stage	References
Inflammation		
IL-1β antibody (Canakinumab) for type 1 diabetes	Clinical trial Phase II	(18)
IL-1β antibody (Canakinumab) for type 2 diabetes	Clinical trial Phase III	(19)
IL-1β antibody (Gevokizumab) for type 2 diabetes	Clinical trial Phase I	(20)
IL-1β antibody (LY2189102) for type 2 diabetes	Clinical trial Phase II	(21)
IL-1β antibody	*In vivo* animal model	(22)
IL-1β receptor antagonist (Anakinra) for type 1 diabetes	Clinical trial Phases II, III	(18)
IL-1β receptor antagonist (Anakinra) for type 2 diabetes	Clinical trial Phase II	(23)
TNFα antibody (Golimumab)	Clinical trial Phase II	(24)
TNFα + T-cell receptor antibodies	*In vivo* animal model	(25)
IL-17 + IL-6 + T-cell receptor antibodies	*In vivo* animal model	(26)
IL-2 antibody	*In vivo* animal model	(27)
IL-21 antibody + Liraglutide	*In vivo* animal model, clinical trial Phase II	(28, 29)
IL-1β receptor antagonist (Anakinra) + CD3 antibody	*In vivo* animal model	(30)
CD3 antibodies	*In vivo* animal model, clinical trial Phase II	(31–34)
Protein kinase (MST1,TAKI,TPL2) inhibitors	*In vitro*, *in vivo* animal models, human islets	(35–39)
Tyrosine kinase (c-Abl,TYK2 JAK1) inhibitors	*In vitro*, *in vivo* animal models, human islets, clinical trial Phase II	(40–43)
Oxydative stress inhibitors	*In vitro*, *in vivo* animal models	(44–50)
HDAC (HDAC3) inhibitors	*In vitro*, *in vivo* animal models	(51–57)
Glucotoxicity and glucolipotoxicity		
Oxydative stress inhibitors and iron chelators	*In vitro*, *In vivo* animal models, human islets	(44–48, 58–60)
Protein kinase (GSK3β, IKKβ, MSTI) inhibitors	*In vitro*, *in vivo* animal models, human islets	(35–37, 61–66)
ER stress inhibitors	*In vitro*, *in vivo* animal models, human islets	(67–71)
TXNIP expression inhibitors	*In vitro*, *in vivo* animal models, clinical trial Phase II	(72–74)
HDAC (HDAC3, HDAC7) inhibitors	*In vitro*, human islets	(75–79)
Triglyceride synthesis (Diacylglycerol acyItransferase 1) inhibitor	*In vitro*, *in vivo* animal models, clinical trial Phase I	(80, 81)
Angiotensin 1 receptor blockers	*In vitro*, *in vivo* animal models, clinical trial Phase III	(82–85)
Adipokine	*In vitro*, *in vivo* animal models, human islets	(86)
Vitamin D	*In vitro*, *in vivo* animal models, clinical trials Phases II, III	(87–95)
Voltage-dependent anion channel-1 inhibitor	*In vitro*, *in vivo* animal models, human islets	(96)
Other inhibitors CK compound, curcumin	*In vitro*	(97–99)
Protectoxicity		
IAAP inhibitory peptide Intravenous immunoglobulin	*In vitro*, *in vivo* animal models	(100–108)
Alphal-antitypsin/Autophagy enhancer/Nanoparticules Nanobodies/Quantum dots/Chaperones/Natural compounds	*In vitro*, *in vivo* animal models	
Senescence		
Bcl2 inhibitor	*In vitro*, *in vivo* animal models	(109)
Hyperglucagonemia		
Glucagon receptor antibodies	*In vitro*, *in vivo* animal models	(110–112)
Glucotoxicity/glucolipotoxicity/proteotoxicity/inflammation		
Approved type 2 diabetes therapeutics	*In vitro*, *in vivo* animal models, human islets	(113–129)

## 2 Inhibition of β-cell dysfunction and death induced by inflammation

β-cell loss caused by immune-mediated processes is a key feature in the pathogenesis of T1D (1–4). Proinflammatory cytokines, including interleukin-1β (IL-1β), tumor necrosis factor-α (TNF-α), and other mediators, are produced and released by immune cells invading the endocrine pancreas and promoting apoptotic death and dysfunction of β-cells (1, 2, 130). Pancreatic islet transplantation turns out to be a promising alternative therapy for some patients with T1D (131, 132). Nevertheless, clinical outcome is not always achieved because of significant loss of islet mass during and after transplantation (132, 133). Up to 80% of transplanted islets die during the post-transplantation period as a result of apoptotic death due to various mechanisms, such as the instant blood-mediated inflammatory response (IBMIR) and the release of various cytokines including IL-1β, TNF-α and IFN-γ (132–134). Chronic, systemic and low grade inflammation was also proposed to lead to β-cell dysfunction and death, and ultimately to T2D (16, 17).

β-cell dysfunction and death induced by cytokines, such as IL-1β, TNF-α, and IFN-γ, occur through stimulation of kinases, mitochondrial dysfunction and death signals, ER stress, and activation of gene transcription factors and regulators. Mitogen-activated protein kinases (MAPKs) (i.e. c-Jun N-terminal kinase (JNK) and p38 MAPK) activated by proinflammatory cytokines trigger the release of mitochondrial death signals and ER stress (1, 2, 135). The transcription factor NFκB is well described as a key mediator of cytokine-induced apoptosis (136, 137). The active transcriptional subunit p65 of NFκB is sequestered and inactivated in the cytoplasm by the inhibitor of NFκB (IκB) under resting conditions. Cytokines, such as IL-1β, induce phosphorylation-dependent degradation of IκB *via* activation of the IκB kinase (IKKβ), which favors the translocation of the active transcriptional subunit p65 to the nucleus and subsequent proinflammatory gene expression. Gene transcription is controlled by epigenetic mechanisms, including DNA methylation or demethylation, and/or histone acetylation or deacetylation. Enzymatic deacetylation of lysine residues within histone is regulated by histone deacetylase (HDAC) enzymes. HDACs were identified as key mediators of β-cell dysfunction and death induced by cytokines (138, 139) (**Figure 1**). Inhibition of these pathways using antibodies, antagonists, or small molecules inhibitors represents a key strategy to prevent cytokine-induced dysfunction and apoptotic death of β-cells for both type of diabetes. Hence, immune-modulatory strategies for T1D therapy, improvement of islet transplantation outcomes, and for T2D therapy have been extensively designed and developed (2, 16, 17, 134, 140) (**Figure 1**).

### 2.1 Cytokine antibodies

Over the last years, cytokine antibodies and combination immunotherapy approaches were designed and tested in their capacity to preserve the β-cell survival and function, and to reduce the progression of diabetes. The cytokine IL-1β was shown to induce β-cell death and dysfunction in both T1D and T2D, and blocking IL-1β deleterious actions using specific IL-1β antibodies was proposed. Randomised, controlled trials of blockade of IL-1β have been performed to investigate any improvement of β-cell function in recent-onset T1D using canakinumab, a human monoclonal anti-IL-1β antibody (18). Canakinumab was reported to be safe but was not effective as single immunomodulatory drug in recent-onset T1D. Authors proposed that IL-1β blockade might be more effective in combination with treatments that target adaptive immunity in organ-specific autoimmune disorders (18). Administration of a custom-made, rat-specific IL-1β monoclonal antibody to Cohen diabetes-sensitive rat, a genetic model of nutritionally induced diabetes when fed a high-sucrose/low-copper diet, counteracted β-cell dysfunction and glucose intolerance (22). Randomised, controlled trial of blockade of IL-1β using canakinumab has been also performed to investigate any improvement on cardiovascular events and T2D. Canakinumab treatment had similar effects on major cardiovascular events among patients with and without diabetes, however treatment did not reduce incident diabetes (19). Other IL-1β antibodies (i.e. gevokizumab, LY2189102) looked promising in several clinical trials for the treatment of T2D (20, 21). However, to date, no treatment for diabetes with anti-IL-1β antibodies has been approved. Despite data from preclinical and clinical studies, this reveals that an improvement in our knowledge is still necessary to develop this type of therapeutic strategy for diabetes.

TNF-α exerts toxic effects on β-cells (141). Patients with new-onset overt T1D generally have elevated serum TNF-α (142). Golimumab, a human monoclonal antibody specific for TNF-α, was tested to determine whether anti-TNF-α antibody could preserve β-cell function in youth with newly diagnosed overt T1D. In a phase 2, multicenter, placebo-controlled, double-blind, parallel-group clinical trial, golimumab treatment resulted in increased insulin production and less exogenous insulin use among children and young adults with newly diagnosed overt T1D (24). Therapy using anti-TNF-α antibody, alone or in combination with an anti-T-cell receptor (TCR) specific antibody was designed to prevent the emergence of T1D. Combination of these two antibodies increased β-cell proliferation, reduced apoptosis leading to a restoration of β-cell mass and function associated with decreased immune cell infiltration in the LEW.1AR1-iddm T1D rat model (25).

The cytokine IL-17A is well known as a key player in autoimmune processes, whereas the cytokine IL-6 is described for maintaining the inflammatory process (143, 144). Therapies with anti-IL-17A or anti-IL-6 antibody in combination with an anti-TCR antibody, or in a triple combination were tested for their capacity to reverse diabetes in the LEW.1AR1-iddm rat. The anti-TCR combination therapy with anti-IL-17 increased the β-cell proliferation and mass, while anti-IL-6 strongly reduced β-cell apoptosis and the islet immune cell infiltrate. The triple combination therapy achieved a complimentary anti-autoimmune and anti-inflammatory action resulting in sustained normoglycaemia (26).

Interleukin-2 was reported to suppress immune pathologies by preferentially expanding regulatory T cells (T_regs_). Notably, administration of an human anti-IL-2 antibody (i.e. F5111.2), which promotes T_reg_ expansion and function selectively, to non-obese diabetic mice (NOD), an animal model of T1D, reduced the progression of diabetes (27).

The cytokine IL-21 was proposed as a suitable target for an immuno-modulatory strategy, and glucagon-like peptide-1 receptor agonists were reported to be appropriate for β-cell protection in the context of T1D. Notably, an anti-IL-21 monoclonal antibody combined with a GLP-1 receptor agonist (i.e. liraglutide) reduced hyperglycemia in NOD mouse model of T1D (28). This combination of anti-IL-21 antibody and liraglutide preserved β-cell function in recently diagnosed T1D patients (29). Efficacy and safety of this combination deserve to be evaluated in a phase 3 trial programme (29).

### 2.2 IL-1β antagonist

The IL-1β receptor antagonist, anakinra, approved to treat rheumatoid arthritis, has been proposed to prevent autoimmune destruction of β-cells in patients with T1D. Randomised, controlled trials of blockade of IL-1β have been performed using anakinra (18). Anakinra was reported to be not effective as single immunomodulatory drug in recent-onset T1D (18). Administration of anakinra was found to exert antidiabetic effects in combination with anti-CD3 antibodies in NOD mice, suggesting that anakinra/anti-CD3 antibody combination therapy may be a potent treatment strategy in patients with T1D (30).

IL-1β receptor antagonist did show promise in clinical trials for the treatment of T2D (23). Treatment with anakinra led to improved glycemia by increasing β-cell function (23). Moreover, exposure to high glucose concentrations induces IL-1β production and secretion from β-cells leading to apoptosis which was prevented by IL-1β receptor antagonist. However, it should be noted that blocking IL-1β signalling by antagonist has shown modest improvement in β-cell function, although the long-term efficacy remains to be determined. It should also be noted that there are still no approved treatment for diabetes that targets innate immune mediators, despite large preclinical and clinical trial data and evidences demonstrating that targeting inflammatory pathways can prevent cardiovascular disease and complications in patients with diabetes (for review see 16, 17).

### 2.3 CD3 antibodies

The signalling hexamer CD3 is a T-cell co-receptor which is described to be essential for the activity of the T-cell receptor. Anti-CD3 monoclonal antibodies bind to CD3 and induce tolerance of Tregs in autoimmune disease. The use of anti-CD3 monoclonal antibodies targeting the autoimmune destruction of β-cells in T1D was evaluated in clinical trials, and has been proposed as potential treatment providing key benefits for patients with T1D (for review see 31, 32). Oral anti CD3-specific monoclonal antibody treatment was reported to induce changes in effector and regulatory T cell compartments, and to reduce incidence of diabetes in NOD mice (33). Fingolimod (FTY720), a sphingosine-1-phosphate receptor modulator, is described to prevent islet damage and to preserve β-cell mass by inhibiting apoptosis and increasing β-cell survival. Combination therapy of an antibody directed against the T cell receptor of the TCR/CD3 complex with fingolimod increased the β-cell mass, blocked islet infiltration, and was effective to reverse T1D in the LEW.1AR1-iddm rat (34).

### 2.4 Protein kinase inhibitors

The serine/threonine kinase mammalian sterile 20-like kinase 1 (MST1) was found to mediate β-cell apoptotic death and dysfunction induced by cytokines (35, 36). MST1 is upregulated in β-cells exposed to cytokines, high glucose concentrations, activates downstream JNK kinases, apoptotic pathways, and impairs insulin secretion through proteasomal degradation of the β-cell transcription factor pancreatic and duodenal homeobox 1 (PDX1) which is critical for insulin production (35). MST1 deficiency restored normoglycemia and β-cell function and prevented the development of diabetes (35). Neratinib, an approved drug targeting HER2/EGFR dual kinases, was identified as a potent inhibitor of MST1, and shown to protect β-cells against the deleterious effects of autoimmune process in T1D (37).

The serine/threonine kinase transforming growth factor-β activated kinase-1 (TAK1, or MAP3kinase 7), a member of the mitogen-activated protein kinase kinase kinase (MAP3K) family, is well described to activate JNK and NFκB signalling pathways, and to be essential in innate and adaptive immune responses. Administration of 5Z-7-oxozeaenol, which inhibits both the kinase and ATPase activity of TAK1, decreased the incidence and delayed the onset of diabetes in NOD mice, reduced insulitis, preserved islet function, and inhibited downstream JNK and NF-κB signalling pathways in pancreatic tissues (38).

Multiple cytokines are likely present simultaneously within islets of T1D patients with powerful synergistic effects (1, 2, 130). IBMIR and the release of various cytokines including IL-1β, TNF-α and IFN-γ during the post-transplantation period destroy the transplanted islets (134). To protect β-cells in T1D and in pancreatic islet transplantation, the identification of a common regulator of β-cell apoptotic death induced by several pro-inflammatory would emerge as a key therapeutic target. Our group has discovered conditions in which targeting the tumor progression locus 2 kinase (TPL2, or MAP3kinase 8), produces potent anti-diabetic effects by preventing dysfunction and apoptosis of β-cells (39). TPL2 is a serine/threonine kinase member of the MAP3K family that stimulates downstream signalling kinases such as JNK and p38 MAPK, and is activated by IL-1β, TNF-α, several chemokines, and toll-like receptor (TLR) ligands. We reported that TPL2 expression was upregulated in β-cells following chronic exposure to cytokines. Pharmacological inactivation of TPL2 using a specific small molecule inhibitor prevented JNK and p38 kinase activation, apoptotic death, and dysfunction of β-cells and human islets induced by IL-1β and mixture of proinflammatory cytokines (IL-1β + TNFα + IFNγ) (39).

Tyrosine-kinase inhibitors have been proposed to prevent T1D progression (40–43). These tyrosine-kinase inhibitors include the approved small molecule kinase inhibitor imatinib which is used to treat chronic leukaemia. Although imatinib inhibits a number of tyrosine kinases, it is quite selective toward the tyrosine kinase c-Abl that is present in various cancers. Imatinib inhibits the c-Abl protein by binding to the ATP pocket in the active site, thus preventing downstream phosphorylation of target protein. c-Abl controls many downstream signalling pathways that are implicated in cellular proliferation, cellular motility, and apoptosis. Treatment of streptozotocin-injected mice with imatinib counteracted diabetes (40). Imatinib decreased the apoptotic death of human islets exposed to cytokines. NFκB signalling was proposed to mediate the antiapoptotic action of imatinib (40). Safety and efficacy of imatinib in preserving β-cell function in patients with recent-onset T1D were evaluated. Importantly, imatinib treatment preserved β-cell function in adults with recent-onset T1D (41). Members of the mammalian Janus kinases (JAK) family (i.e. JAK1, JAK2, JAK3 and tyrosine kinase 2 (TYK2)) have been shown to mediate inflammation in β-cells. Pharmacological inhibitors of TYK2 were developed using structure-based drug design. Inhibitors were found to bind to the JH2 pseudokinase domain of TYK2 and to act as allosteric inhibitors of the kinase activity. The JH2-directed allosteric inhibition provides the high specificity for TYK2 over other JAK family members. These TYK2 inhibitors were reported to protect human β-cells against the deleterious effects of IFNγ (42). A JAK1-selective inhibitor, ABT 317, was found to reduce IL-21, IL-2, IL-15 and IL-7 signalling in T cells, and IFN-γ signalling in β cells. Notably, diabetes was reversed in mice treated with ABT 317 (43).

### 2.5 Oxydative stress inhibitors

Proinflammatory cytokines induce the formation of ROS. β-cells are susceptible to formation of ROS because of low expression of anti-oxidative enzymes such as catalase, superoxide dismutase and glutathione peroxidase. Increased ROS formation induces β-cell damages (for review see 44–48). As example, **e**xcessive production of ROS by the phagocyte-like NADPH oxidase2 (Nox2) drives β-cells toward oxidative damages. Rac1, a small G-protein, is one of the members of Nox2 holoenzyme. NSC23766, a known inhibitor of Rac1, significantly attenuated cytokine-induced Nox2 activation and ROS formation in β-cells. Administration of NSC23766 to diabetic NOD mice suppressed Rac1 expression and activity, the endoplasmic reticulum stress, and prevented the development of diabetes (49).

The potential beneficial effects of hesperidin in T1D, a flavanone glycoside present in all citrus fruits, were investigated. Hesperidin exerted beneficial effects on β-cells reducing oxidative stress by increasing antioxidant SOD and GPx activities, decreasing nitrotyrosine and malondialdehyde levels, upregulating of anti-apoptotic Bcl-xL, downregulating of pro-apoptotic Bax and cleaved caspase-3. Treatment of streptozotocin diabetic rats with hesperidin reduced hyperglycemia, and increased serum and pancreatic insulin levels. In addition, concentrations of TNF-α and expressions of ER stress makers GRP78 and CHOP proteins were found diminished in the pancreas following the hesperidin treatment (50).

### 2.6 Histone deacetylase inhibitors

HDACs were proposed as key targets to develop therapeutics for T1D and T2D (for review see 138). Especially, HDACs 1 and 3 were identified as key mediators of β-cell cytotoxicity and death (138, 139). Specific knock-down of HDAC3 reduced binding of the NFκB subunit p65 to DNA and consequently reduced inflammatory gene transcription (139). HDAC inhibitors are natural products or synthetically produced, include pan-HDAC inhibitors and class-selective or isoform-selective inhibitors. The mechanisms of action of HDAC inhibitors are still unclear. However, these inhibitors are emerging therapeutic agents that have been clinically validated in cancer patients with hematologic malignancies.

Several HDAC inhibitors were reported to reduce β-cell apoptosis induced by proinflammatory cytokines (51–53). Vorinostat is the most advanced pan-HDAC inhibitor and was approved for the treatment of patients with hematologic malignancies including cutaneous T-cell lymphoma. Givinostat prevented β-cell apoptosis and expression of the β-cell toxic inflammatory molecule IL-1β (53). Sodium butyrate (NaB) is a short chain fatty acid having HDAC inhibition activity. NaB treatment decreased plasma glucose, improved plasma insulin level and glucose homeostasis through HDAC inhibition in streptozotocin injected-diabetic animal. NaB treatment was found to improve β-cell proliferation and function, and to reduce β-cell apoptosis by the modulation of p38/ERK MAPK and apoptotic pathway (54). Administration of BRD3308, an inhibitor of HDAC3, to NOD mice preserved β-cell function and survival, enhanced β-cell proliferation, decreased infiltration of mononuclear cells in islets, and reduced the emergence of diabetes (55).

Combination of the orally active form of givinostat with humanised CD3 antibodies (otelixizumab) synergised to prevent islet inflammation, to improve β-cell function and survival reducing diabetes in NOD mice (56). Combination of HDAC inhibitor vorinostat with the dipeptidyl peptidase-4 (DPP-4) inhibitor MK-626 increased β-cell mass without preventing diabetes. Authors proposed the combination of vorinostat and MK-626 as a beneficial adjunctive therapy in clinical trials for T1D prevention or remission (57).

## 3 Inhibition of β-cell dysfunction and death induced by glucotoxicity and glucolipotoxicity

T2D is characterized by a gradual loss of β-cell function and mass (5–10). It is now well described that nutrient-induced metabolic stresses, such as chronic hyperglycemia, are crucial factors underlying deterioration of β-cell function and mass. We and others have shown that chronic exposure of β-cells to hyperglycemia deteriorates insulin secretion and induces apoptosis (11–14, 145, 146). Studies *in vitro* provided evidences for synergistic adverse effects of elevated glucose and saturated fatty acid high concentrations on the function and survival of β-cells (for review see 14, 147, 148). High levels of glucose and fatty acids such as palmitate (alone or in combination) induce insulin secretion dysfunction and apoptosis in β-cells (i.e. glucotoxicity, lipotoxicity and glucolipotoxicity) through mitochondrial alteration, ROS formation, stimulation of kinases, ER stress, activation of HDACs, inflammation, alteration of protein degradation pathways and impaired autophagy (14, 15, 147–149) (**Figure 2**). Inhibitions of these pathways are key strategies to prevent β-cell dysfunction and apoptosis.

### 3.1 Oxydative stress inhibitors and iron chelators

Chronic exposure of β-cells to high glucose leads to increased ROS formation (for review see 44–48). The β-oxidation of saturated non-esterified fatty acids in peroxisomes and mitochondria generate hydrogen peroxide. Several antioxidants were reported to protect β-cells against ROS-induced damages (44–48). As an example, a major flavanone glycoside in citrus species, naringin, was found to decrease ROS accumulation, and to exert protective effects on islet dysfunction and diabetes by amelioration of hyperglycemia (150).

The tumor suppressor p53 protein is activated by oxidative stress to induce mitochondrial dysfunction and has been proposed to play a role in T2D development. p53 deficiency protected against diabetes in streptozotocin-induced diabetes and *db/db* mouse. Moreover, treatment of *db/db* mouse with pfithrin-α, a reversible inhibitor of p53-mediated apoptosis and p53-dependent gene transcription, improved mitochondrial dysfunction and glucose intolerance (58).

Iron is a redox-reactive metal which can catalyse ROS formation. Increased expression of divalent metal transporter 1 correlates with increased β-cell iron content and ROS formation. Notably, knockout mice for the divalent metal transporter 1 were found to be protected against streptozotocin and high-fat diet-induced glucose intolerance (59). Iron chelators deferoxamine and deferasirox are approved drugs for the treatment of acute iron toxicity and transfusion-induced iron overload. Deferoxamine and deferasirox were shown to reduce ROS formation and β-cell apoptosis. Notably, increased levels of the iron-carrying plasma protein transferrin was reported to be associated with a risk of developing both type of diabetes (60).

### 3.2 Protein kinase inhibitors

Glycogen synthase kinase-3 (GSK3) is a ubiquitously expressed, highly conserved serine/threonine protein kinase. GSK3 is involved in various signalling pathways regulating glycogen metabolism, cell development, gene transcription, protein translation, cytoskeletal organization, cell cycle regulation, proliferation, and apoptosis. The role of GSK3β was particularly studied in pathways activated in β-cells and for the treatment of diabetes. Studies reported the mood stabilizer valproate (valproic acid) as an inhibitor of GSK-3β activity. In INS-1 β-cell line, valproate protected cells to palmitate-induced apoptosis (61). An inhibitor of GSK-3β, TDZD-8, was shown to exert same anti-apoptotic effect (61). Administration of KICG1338, another inhibitor of GSK-3β, preserved β-cell function *in vivo* (62). Using high-sensitivity mass spectrometry-based proteomics analysis, Sacco and colleagues confirmed that GSK3β is a key kinase involved in signalling network controlling the β-cell-specific transcription factor PDX1. GSK3β was found to mediate the insulin secretion dysfunction induced by chronic hyperglycemia. Pharmacological inhibition of GSK3β restored the ability of β-cells to secrete insuline in response to glucose (63). Treatment of Goto-Kakizaki rats, a spontaneous nonobese model that develops T2D, with infra-therapeutic doses of lithium, a widely used inhibitor of GSK3, reduced the expression of pro-inflammatory cytokines in the pancretic islets, partially restored the glucose-induced insulin secretion, and reduced the development of diabetes (64). GSK3β was further found to mediate the pro-apoptotic effects of glucocorticoids in β-cells (65).

A key role for IKKβ in β-cell dysfunction induced by fatty acids was demonstrated (66). An inhibitor of IKKβ, BMS-345541, and deletion of IKKβ prevented β-cell dysfunction *in vitro* and *in vivo* (66).

The serine/threonine kinase MST1 was found to be a key regulator of β-cell apoptotic death and dysfunction induced not only by cytokines but also by chronic hyperglycemia (35, 36). Administration of neratinib, a potent inhibitor of MST1, improved β-cell survival and preserved β-cell mass and function in *db/db* mouse model of T2D (37).

### 3.3 Endoplasmic reticulum stress inhibitors

The ER stress has been reported to play an important role in β-cell dysfunction and death in the context of T2D (for review see 67–69). Restoring ER homeostasis, enhancing ER-associated degradation of misfolded protein, and boosting chaperoning activity are proposed as therapeutic approaches for the treatment of diabetes (67–70). Over the last years, reviews exhaustively listed inhibitor molecules existing against ER stress and tested to protect β-cells against diabetogenic conditions. Several inhibitors exist for each of the major molecules involved in ER stress such as IRE1, PERK and ATF6. As examples, imatinib, kinase-inhibiting RNase-attenuators 6 (KIRA6) and KIRA8 are three IRE1 inhibitors. GSK2606414 and GSK2656157 are two PERK inhibitors. Nelfinavir and ceapins are two ATF6 inhibitors which exert protective effects on β-cell function and mass against ER stress (for review see 67–70).

Tauroursodeoxycholic acid is a taurine conjugate of ursodeoxycholic acid, naturally occurring hydrophilic bile acid, and approved for primary biliary cholangitis treatment. Tauroursodeoxycholic acid was reported to exert therapeutic benefits in various models of diseases such as diabetes, obesity, and neurodegenerative diseases, due to its cytoprotective effect. The mechanisms underlying this cytoprotective effect have been attributed to reduction of ER stress and stabilization of the unfolded protein response, which contributed to naming tauroursodeoxycholic acid as a chemical chaperone. This chemical chaperone decreased palmitate-induced apoptosis in β-cells by reducing the expression of CHOP and ATF4 involved in ER stress (71).

### 3.4 TXNIP expression inhibitors

Thioredoxin-interacting protein (TXNIP), a member of the alpha arrestin protein family, has emerged as a major factor regulating β-cell dysfunction and death in the pathogenesis of T2D and T1D (72–74, 151–154). Thioredoxin is a thiol-oxidoreductase and a major regulator of cellular redox signalling which protects cells from oxidative stress. TXNIP inhibits the antioxidative function of thioredoxin resulting in ROS accumulation. TXNIP also regulates the mitochondrial death pathway and ER stress. TXNIP expression was found to be up-regulated in diabetogenic conditions. Exposure to high glucose upregulates TXNIP expression in β-cells (152). TXNIP plays a key role in mediating the detrimental effects exerted by glucotoxicity. TXNIP promotes β-cell apoptosis, inhibits insulin production and β-cell function. Whole body TXNIP-deficient and β-cell-specific TXNIP knockout mice have decreased β-cell apoptosis, increased β-cell mass, elevated insulin levels, and are protected from diabetes (for review see 153). Exposure to inflammatory cytokines such as IFN-γ and cytokine mixture (i.e. IL-1β + TNF-α + IFN-γ) was also found to increase TXNIP expression in β-cells by distinct mechanisms (154).

Following a high-throughput screen, an orally bioavailable, non-toxic small molecule, SRI-37330, was found to effective in inhibiting TXNIP expression. In human islets, SRI-37330 inhibited TXNIP expression. SRI-37330 treatment rescued mice from obesity (*db/db*) - and streptozotocin-induced diabetes (72). In addition, SRI-37330 treatment inhibited glucagon secretion and function, reduced hepatic glucose production, and reversed hepatic steatosis (72).

The approved antihypertensive calcium-channel blocker, verapamil, was reported to promote β-cell survival by decreasing the expression of TXNIP. Verapamil is believed to decrease β-cell TXNIP expression by reducing cytosolic calcium, which in turn controls calcineurin/calcium-dependent protein phosphatase 2B signalling and leads to increased phosphorylation and nuclear exclusion of carbohydrate response element-binding protein (ChREBP) and results in decreased ChREBP mediated TXNIP transcription. Oral administration of verapamil reduced TXNIP expression and β-cell apoptosis, enhanced endogenous insulin levels, protected BTBR *ob/ob* mice as a model of T2D and mice from streptozotocin-induced diabetes (73). Retrospective studies suggest that verapamil use might be associated with a lower incidence of T2D in humans (for review see 153). A randomized double-blind placebo-controlled phase 2 clinical trial was further designed and conducted to assess the efficacy and safety of oral verapamil added to a standard insulin regimen in adult individuals with recent-onset T1D. Notably, addition of once-daily oral verapamil was shown to be a safe and effective strategy to promote β-cell function and to reduce insulin requirements in adult individuals with recent-onset T1D (74).

### 3.5 Histone deacetylase inhibitors

The potential therapeutic effect of HDAC3 pharmacologic inhibition on T2D has been particularly investigated. RGFP966 (HDAC3 inhibitor) enhanced insulin secretion and synthesis in normal and diabetic mice islets and reduced partially palmitate-induced apoptosis in NIT-1 cells (75). We reported that MS-275 (HDAC3 inhibitor) prevented β-cell death induced by palmitate in human pancreatic islets (76). MS-275 was further found to potentiate insulin secretion (77). Other HDAC inhibitor such as sodium butyrate prevented β-cell dysfunction and death in diabetic rat and mice (78). Notably, the HDAC7 inhibitor MC1568 was reported to protect β-cells from dysfunction and death, suggesting that specific inhibitors for HDAC7 may be useful for T2D treatment (79).

### 3.6 Triglyceride synthesis inhibitor

Diacylglycerol acyltransferase 1 (DGAT1), using for the triglyceride synthesis, has been reported as a potential therapeutic target. DGAT1 inhibitor improved palmitate-induced apoptosis in β-cells and mice pancreatic islets. DGAT1 inhibitor was further found to protect β-cells against inflammation and endoplasmic reticulum stress. DGAT1 inhibitor treatment in *db/db* mice decreased hyperglycemia, triglyceride levels, and improved glucose tolerance (80).

Inhibition of DGAT1 was evaluated as a potential treatment modality for patients with obesity and T2D. A randomized, phase 1 study in overweight or obese men explored the effects and tolerability of AZD7687, a reversible and selective DGAT1 inhibitor. Dose-dependent reductions in postprandial serum triacylglycerol were demonstrated with AZD7687 treatment. However, gastrointestinal side effects were observed and several participants discontinued treatment due to diarrhea making the usefulness of DGAT1 inhibition as a novel treatment for diabetes and obesity questionable (81).

### 3.7 Angiotensin receptor blockers

In a large clinical prospective trial, inhibition of the renin-angiotensin system was found to delay the onset of T2D in high-risk individuals (82). Losartan, a selective angiotensin 1 receptor (AT1R) blocker, was reported to protect human islets against glucotoxicity by inhibiting increased ER stress markers such as GRP78, sXBP1, ATF4 and Grp78. Losartan treatment improved insulin secretion (83). Telmisartan, another AT1R blocker, is a common drug used for hypertension treatment. Telmisartan was found to exert protective effects against high glucose/high lipid-induced β-cell apoptosis and to improve the insulin secretion by inhibiting oxidative and ER stress (84). Treatment of *db/db* mice with combination of telmisartan and the DPP-4 inhibitor linagliptin preserved islet cell function and morphology *via* reduction of oxidative stress (85).

### 3.8 Adipokine

The adipokine adipsin/complement factor D controls the alternative complement pathway and generation of complement component C3a, and was shown to increase insulin secretion. Higher concentrations of circulating adipsin were associated with a significantly lower risk of developing future diabetes among middle-aged adults. Administration of adipsin in *db/db* mice was found to improve hyperglycemia, to increase insulin levels, and to preserve β-cell function by blocking dedifferentiation and death. Adipsin/C3a was further found to decrease the phosphatase Dusp26 which negatively controls the expression of core β-cell identity genes and sensitizes cell to apoptotic death. Pharmacological inhibition of Dusp26 improved hyperglycemia in *db/db* mice and protected human islet cells from apoptotic death (86).

### 3.9 Vitamin D receptor/BRD9 association inhibitor

Impaired β-cell function has been reported with low blood 25-hydroxyvitamin D levels (87). Epidemiological and human genetic studies revealed a link between vitamin D and the vitamin D receptor (VDR) to both T1D and T2D (88). The VDR can bind to bromodomain-containing protein 9 (BRD9 protein). Wei Z and colleagues discovered that the pharmacological inhibition of BRD9 promotes the activation of a VDR-dependent transcriptional program that underlies β-cell survival. Activation of the VDR signalling by a synthetic ligand in combination with the BRD9 inhibitor partially restored β-cell function and glucose homeostasis in *db/db* mice and in mice treated with low doses of streptozotocin (89).

A low blood 25-hydroxyvitamin D level has emerged as a possible risk factor for T2D, and vitamin D supplementation has been proposed as a potential intervention to reduce diabetes risk (90, 91). In a multicenter, randomized, placebo-controlled trial involving persons at high risk for T2D not selected for vitamin D insufficiency, vitamin D_3_ supplementation did not result in a significantly lower risk of diabetes (92). In comparison, the Tromsø Vitamin D and T2DM Trial, which randomly assigned adults with prediabetes to vitamin D_3_ or placebo, the risk of diabetes was numerically lower in the vitamin D group than in the placebo group, but the difference was not significant (93). In other clinical trials which randomly assigned adults with prediabetes to an active form of vitamin D analogue (eldecalcitol) or placebo, the risk of diabetes was also lower in the vitamin D group than in the placebo group, but the difference was again not significant (94, 95).

### 3.10 Voltage-dependent anion channel-1 inhibitor

The ATP-conducting mitochondrial outer membrane voltage-dependent anion channel-1 (VDAC1) was found to be upregulated in pancreatic islets of T2D organ donors. VDAC1 overexpression causes its mistargeting to the plasma membrane of the β-cells with loss of the metabolic coupling factor ATP. Through direct inhibition of VDAC1 conductance, metformin and specific VDAC1 inhibitors (VBIT-4) and antibodies were reported to restore the impaired generation of ATP and glucose-stimulated insulin secretion in T2D pancreatic islets. Treatment of *db/db* mice with VDAC1 inhibitor VBIT-4 prevented hyperglycemia, and maintained β-cell function and glucose tolerance (96).

### 3.11 Other inhibitors

Compound K (CK) has been described to possess anti-diabetic properties, but the mechanism of action is still not clear. Treatment of diabetic mice with CK decreased fasting plasma glucose, triacylglycerol, total cholesterol, elevated plasma insulin levels and improved glucose tolerance. CK treatment inhibited β-cell apoptosis and caspase-3 activity with a decrease in JNK activation (97).

The phytochemical curcumin decreased NAPDH oxidase and apoptotic factor expression induced by high glucose and palmitate involved in pancreatic islet death (98). Curcumin was further reported to protect RIN-m5F β-cells against apoptotic damages induced by oxidative stress (99). The mechanism of action of curcumin in β-cells is still not fully elucidated.

## 4 Inhibition of β-cell dysfunction and death induced by proteotoxicity

Amyloid deposition derived from amyloid polypeptide (IAPP), a protein which is synthesized and secreted with insulin by β-cells, are frequently found in pancreatic islets of T2D patients (155, 156). IAPP forms oligomers and subsequently insoluble fibrils in species at risk of developing diabetes (157–159). Supporting a role of IAPP in the development of T2D in humans, a rare missense mutation in the IAPP gene (S20G) that increases its amyloidogenicity has been reported to be associated with β-cell failure and the development of T2D (160, 161). In rodents, IAPP is well known to be nonamyloidogenic. Overexpression of human IAPP (hIAPP) in rodent models promotes amyloid deposits, β-cell dysfunction and apoptosis, leading to reduced β-cell functional mass and hyperglycemia (162, 163). IAPP induces β-cell dysfunction and apoptosis by promoting dysruption of mitochondrial network dynamics, oxidative stress, ER stress, defects in pathways regulating protein degradation, impairment of autophagy and inflammation (for review see 15, 155, 156, 164). The effects of drugs currently used for the treatment of T2D, hIAPP mimetics and peptides, small organic molecules, natural compounds, nanoparticles, nanobodies, quantum dots, metals and metal complexes, and chaperones found to potentially inhibit and/or reverse hIAPP aggregation have been extensively reviewed (100). As examples, we can cite an IAPP inhibitory peptide, an intravenous immunoglobulin, an alpha1-antitrypsin, and an autophagy enhancer.

### 4.1 IAPP inhibitory peptide

Self-assembly into oligomers and fibrils during the process of aggregation by hIAPP can lead to β-cell failure. Some critical regions of hIAPP might contribute to the aggregation, and finding effective molecules, especially short-peptide inhibitors that bind to these regions and disrupt the aggregation of hIAPP are of interest. The IAPP inhibitory peptide, D-ANFLVH, was shown to prevent islet amyloid accumulation in cultured human islets. This D-ANFLVH peptide was administered into the hIAPP overexpressing transgenic mouse model, and found to be a potent inhibitor of islet amyloid deposition, resulting in decreased islet cell apoptosis and preservation of β-cell area leading to improved glucose tolerance (101).

### 4.2 Intravenous immunoglobulin

Intravenous immunoglobulin (IVIg) is described to be an efficient anti-inflammatory and immunomodulatory agent for the treatment of several autoimmune or inflammatory neurological diseases. IVIg treatment in hIAPP transgenic mouse model significantly improved glucose control and insulin sensitivity, prevented β-cell apoptosis by lowering toxic IAPP oligomer levels, attenuating islet inflammation and activating autophagy (102).

### 4.3 Alpha1-antitrypsin

Alpha1-antitrypsin (AAT) is a circulating protease inhibitor with anti-inflammatory properties. AAT treatment in mice overexpressing hIAPP in β-cells improved glucose tolerance and restored the insulin secretory response to glucose. AAT prevented the formation of amyloid deposits and apoptosis induced by high glucose concentrations. Notably, AAT protected β-cells against the cytotoxic effects of conditioned medium from hIAPP-treated macrophages. AAT also prevented the cytotoxic effects of proinflammatory cytokines on β-cells, and protected β-cells from the cytotoxic actions of hIAPP mediated by macrophages (103).

### 4.4 Autophagy enhancer

Autophagy is crucial for clearance of hIAPP oligomer, suggesting that an autophagy enhancer could be a therapeutic modality against human diabetes with amyloid accumulation. A recent identified autophagy enhancer (MSL-7) was found to reduce hIAPP oligomer accumulation in human induced pluripotent stem cell-derived β-cells and oligomer-mediated apoptosis of β-cells. MSL-7 administration improved glucose tolerance and β-cell function of hIAPP transgenic mice on high-fat diet, associated with reduced hIAPP oligomer/amyloid accumulation and β-cell apoptosis (104).

### 4.5 Other inhibitors

Studies have showed that selenium-containing phycocyanin inhibited the fibrillation of hIAPP to form nanoscale particles (105, 106). Anti-amyloidogenic potential of azadirachtin, a metabolite isolated of medicinal plant, acts on hIAPP in β-cells. Azadirachtin reduced oxidative and ER stresses with an improvement of glucose-stimulated insulin secretion (107). Chitosan oligosaccharides (COS) have been reported to exhibit a potential antidiabetic effect. COS have the capacity to diassembled preformed hIAPP and inhibited its aggregation. COS reduced IAPP-induced apoptosis in β-cells (108).

## 5 Inhibition of β-cell dysfunction and death induced by senescence

A subset population of β-cells was observed and reported to acquire a senescence-associated secretory phenotype during T1D development in diabetic NOD mice and humans. Senescent β-cells displayed upregulation of the pro-survival mediator Bcl-2. Notably, treatment of NOD mice with a Bcl-2 inhibitor selectively eliminated these cells without altering the abundance of the immune cell types involved in the disease. Elimination of senescent β-cells prevented immune-mediated β-cell destruction and diabetes, suggesting that clearance of senescent β-cells could be an innovative therapeutic approach for T1D (109).

## 6 Inhibition of β-cell dysfunction and death induced by hyperglucagonemia

Glucagon is a peptide hormone secreted from the α-cells of the islets of Langerhans in response to hypoglycemia. Glucagon exerts its intracellular effects through binding to its specific receptor (Glucagon receptor, GCGR) that spans the plasma membrane (for review see 165, 166). The GCGR belongs to the class II (or B) secretin/glucagon/vasoactive intestinal peptide superfamily of 7-transmembrane receptor guanine nucleotide-binding protein (G-protein) coupled receptors (GPCRs) (165, 166). The GCGR is expressed in liver, adipose tissues, kidney, gastrointestinal tract, brain, heart, and β-cells (165, 166). Glucagon increases glucose production and lipid oxidation in the liver, energy expenditure, glomerular filtration in the kidney, reduces motility in the gastrointestinal tract and food intake (165). Hyperglucagonemia is present in all forms of diabetes. Despite controversy regarding the role of glucagon in metabolic disorders associated with diabetes, the recognized importance of hyperglucagonemia in the pathophysiology of T1D and T2D fostered the development of therapeutic strategies aimed at glucagon action reduction (165, 167). Increasing studies propose that blocking glucagon and/or GCGR can reduce hyperglycemia (for review see 165, 167).

Anti-glucagon receptor antibody treatment has been proposed and tested to reverse T1D. Blockade glucagon action using a monoclonal antibody of the GCGR (Ab-4) was found to improve glycemia (110). Notably, treatment with this GCGR monoclonal antibody promoted β-cell survival, and enhanced formation of functional β-cell mass and production of insulin-positive cells from α-cell precursors in NOD mice (110). An antidiabetic effect and a β-cell regenerative action using GCGR antibody were recently confirmed (111). When combined with the immune modulator anti-CD3 teplizumab, GCGR antibody efficiently increased β-cell mass and reversed diabetes in NOD mice (111). GCGR antagonism ameliorated hyperglycemia and promoted β-cell regeneration in *db/db* mice and high-fat diet and streptozotocin-induced mice with T2D (112). The liver-derived fibroblast growth factor 21 (FGF21) was shown to be involved in the GCGR antagonism-induced β-cell regeneration (112).

## 7 Inhibition of β-cell dysfunction and death by approved T2D therapeutics

Several drugs currently used for the treatment of T2D have been reported to protect β-cell function and survival against glucotoxicity, lipotoxicity, proteotoxicity, and inflammation in preclinical studies (113–129).

### 7.1 Metformin

Metformin has been shown to protect rat and human islets from oxidative and ER stress, metabolic dysfunction, apoptosis induced by glucotoxicity and lipotoxicity (113–116). Metformin was further reported to promote INS-1 β-cell proliferation and survival by activating AMPK/SIRT1/PGC-1α signalling pathway and inducing autophagy in high-glucose environment (117). Metformin was found to restore the impaired generation of ATP, through inhibition of VDAC1 conductance, and glucose-stimulated insulin secretion in T2D pancreatic islets (96).

### 7.2 Thiazolidinediones

Thiazolidinediones are small molecules that activate the nuclear receptor PPARγ to increase insulin sensitivity. The potential direct effects of thiazolidinediones on β-cells have been controversial. However, rosiglitazone or pioglitazone were shown to protect β-cells against proinflammatory cytokines (118). Pioglitazone treatment in *db/db* mice protected β-cell function (119). A new thiazolidinedione, lobeglitazone, was found to exert beneficial effects on β-cell function and survival in *db/db* mice (120). Thiazolidinedione treatment may be a promising approach to preserve β-cell mass and function by inhibiting islet amyloid formation and decreasing ER stress induced by hIAPP. Rosiglitazone was recently reported to improve the survival of INS-1E cells exposed to hIAPP. Rosiglitazone inhibited hIAPP fibrillation and decreased hIAPP-induced expression of C/EBP homologous protein (CHOP) (121).

### 7.3 Glucagon-like peptide-1 and gastric inhibitory polypeptide

GLP-1 and GIP are the two primary incretin hormones secreted from the intestine on ingestion of glucose or nutrients to stimulate insulin secretion from β-cells. GLP-1 and GIP exert their actions by binding to the GLP-1 receptor (GLP-1R) and GIP receptor (GIPR), respectively, two receptors that belong to the GPCR family (for review see 122–124). GLP-1 receptor agonists are successfully used for the treatment of T2D (122–124). Using β-cell lines, mouse, rat, human islets, and animal models of T2D, GLP-1 receptor agonists were widely reported to improve β-cell function, to protect β-cells against glucotoxicity, lipotoxicity, and inflammation (for review see 15, 122–124).

### 7.4 Dipeptidyl peptidase-4 inhibitors

DPP-4 inhibitors work by blocking the action of DPP-4, an enzyme that destroys the incretins GLP-1 and GIP. Studies on β-cell lines and human islets have shown that DPP-4 inhibitors protected β-cells against cytokine-induced apoptosis and increased glucose-stimulating insulin secretion (125).

### 7.5 Sodium-glucose co-transporter type 2 inhibitors

SGLT2 are a class of prescribed medicines that are approved for use with diet and exercise to lower blood sugar in adults with T2D. SGLT2 inhibitors lower blood sugar by causing the kidneys to remove sugar from the body through the urine. Dapagliflozin treatment was shown to reduce hyperglycemia in *db/db* mice associated with an increase of β-cell mass (126). Dapagliflozin was also found to be effective to protect β-cell survival in *db/db* mice (127). Empagliflozin has been studied in Zucker diabetic fatty rat model and was found to preserve β-cell function and mass (128, 129).

## 8 Conclusion and perspectives

The European Medicine Agency and the Food Drug Agency recall that there is an unmet and urgent medical need for therapies that act on the pathogenesis of the disease, rather than on the symptoms, and prevent dysfunction and death of β-cells. Treatment strategies focus on insulin replacement therapy (T1D) (4), reducing insulin resistance and preserving insulin secretion (T2D) (7, 8). None of the approved anti-diabetic treatments are effective in protecting functional β-cell mass against diabetogenic stressors. The research has made interesting progress in identifying multiple types of immune cells, soluble factors, and signalling pathways that destroy insulin-producing β-cells. This knowledge produced in recent years on the pathogenesis of the disease has made it possible to propose the development of innovative therapies to prevent and modify T1D and T2D.

As described in this review, a large number of antibodies, receptor antagonists, natural compounds or small molecules inhibitors with diverse chemical structures have been validated both *in vitro* and *in vivo* experimental models to potentially treat T1D and T2D by inhibiting β-cell dysfunction and death. Nevertheless, these antibodies, antagonists, natural compounds or small molecules inhibitors are need to be evaluated for pharmacokinetic properties, toxicity, and efficacy. Clinical trials in humans will be also required to assess the relevance of some of these pharmacological agents as a therapeutic strategy for assessing the safety, dose and/or durability of treatment. A major concern in therapeutic strategies based on the use of pharmacological inhibitors is the high risk of serious side effects, by inhibiting ubiquitously expressed targets and targets with pleiotropic properties. The specificity and selectivity of pharmacological inhibitors in a physiological system have to be studied and represent fundamental informations. However, it should be noted that the use of tyrosine kinase inhibitors and HDAC inhibitors in some cancer treatment is a feasible and promising approach. Another important aspect to consider is the translation of pre-clinical data into clinical trials, which may lead to negative results, as shown by the clinical trials using an IL-1β blockade strategy.

A key question that remains to be solved *Is a specific therapeutic strategy to preserve the function and survival of β cells sufficient to prevent diabetes?* A functional β-cell mass is essential in preventing the development of T1D and T2D. Theoretically, a therapeutic strategy targeting β-cells seems appropriate, but targeting multiple pathways in combination therapies. The release of a new antidiabetic medicine targeting both GLP-1R and GIPR (168) supports the idea that the next antidiabetic therapy for the future management of diabetes could involve the combination of pharmacological agents with complementary mechanisms of action.

## Author contributions

SD, ER, AA wrote the manuscript. All authors contributed to the article and approved the submitted version.
